# Correlation between allergic rhinitis or hay fever and lung cancer: A systematic review and meta-analysis

**DOI:** 10.1097/MD.0000000000038197

**Published:** 2024-05-17

**Authors:** Qudsia Umaira Khan, Muneeb Ur Rehman, Mohammad Ali Arshad Abbasi, Rubina Rafique Shiekh, Munazza Nazir, Sohail Khan Raja, Amna Akbar, Sabahat Tasneem, Sarosh Khan Jadoon, Sarosh Alvi

**Affiliations:** a CMH Lahore Medical College, Lahore, Pakistan; b Lady Reading Hospital, Peshawar, KPK, Pakistan; c Medicine AJKMC, Muzaffarabad, Pakistan; d AJKMC, Muzaffarabad, AJK, Pakistan; e CHPE Health Services Academy, Islamabad, Pakistan; f Health Services Academy, Islamabad, Pakistan; g Combined Military Hospital, Muzaffarabad, Pakistan; h University of Khartoum, Khartoum, Sudan.

**Keywords:** allergic rhinitis, allergy, cancer, hay fever, lung cancer, meta-analysis

## Abstract

**Background:**

The association between allergies and cancer is contradictory, whereas some forms of cancer have inverse associations with allergies. Allergic rhinitis (AR) is the most prevalent form of allergy, and lung cancer is one of the most prevalent forms of cancer with the highest mortality rate. Recent studies have reported a positive association between asthma and lung cancer; however, this association is inconclusive. Furthermore, AR is positively associated with asthma; therefore, our research question was to explore whether there is any correlation between AR and lung cancer epidemiologically.

**Methods:**

After a rigorous search of PubMed, Google Scholar, and ScienceDirect, 7 eligible articles were included in this systematic review and meta-analysis, including 4724 cases and 9059 controls, 5 from the USA, and one each from Canada and Germany.

**Results:**

Pooled analysis (OR, 0.55; 95% CI: 0.45–0.68; *P* value < .00001) showed a strong inverse relationship between AR and lung cancer.

**Conclusion:**

The current meta-analysis suggests an inverse relationship between AR and lung cancer; however, new epidemiological studies are required to observe the current scenario more comprehensively.

**Key Points**

- There are several experimental evidences that a higher IgE level could reduce the probability of cancer.
- Epidemiological studies also reported an inverse association between allergy and cancer of several types.
- AR is characterized by a higher IgE level, and our studies also represent a strong protective association between AR and lung cancer.
- It is essential to gain pathophysiological insights into whether AR plays a role in the prevention of lung cancer. As no population-based studies were found after 2003 after following a broadened search strategy in our study, recent epidemiological studies across different parts of the world are necessary to validate our findings.

## 1. Introduction

Allergies and cancer are continuously increasing health complications in both developing and developed countries. According to a report by the World Allergy Organization, almost one-fifth of the population of developed countries suffer from at least one form of allergy, and the assumption of incidence is even higher in developing countries. Nearly 400 and 300 million people are estimated to be victims of 2 leading forms of allergy globally; allergic rhinitis (AR) and asthma, respectively.^[1,2]^ According to Global Cancer Statistics (GCS) 2020, almost 19.3 million new cancer cases were diagnosed in 2019, and 10.0 million deaths occurred due to cancer. Cancer is debated as the first or second leading cause of mortality. Cancer incidence is also expected to rise; a 47% increase in cases is expected in 2040 compared to 2020.^[3]^ The high global burden of both allergies and cancer has led scientists to consider whether there is any correlation between these 2 conditions.

Lung cancer was the leading form of cancer as per GCS 2018 and was the second leading form of cancer after female breast cancer as per GCS 2020, and the mortality rate was significantly higher in each report compared to any other site of cancer.^[3,4]^ A study was conducted to clinically observe the correlation between different forms of allergy and cancer; however, an inverse association was reported for most types, a contradictory relationship was demonstrated for some forms of cancer, including lung cancer, and a positive association was reported for bladder cancer, prostate cancer, lymphoma, and myeloma.^[5]^ Another study also reported an inverse association between different forms of allergy and cancer; however, there was a clinically positive association between asthma and lung cancer, and atopic dermatitis and skin cancer.^[6]^

Arguably, AR is the most prevalent form of allergy; notably, this percentage is higher among teenagers. Studies in different countries have shown a prevalence of AR ranging from 11.8% to 46.0%.^[7]^ It is a form of inflammation that affects the nasal mucosa, which affects the quality of life, sleep, and work performance, resulting in frequent sneezing, itchy and watery nose, eyes, mouth, and throat.^[8]^ AR is more likely to be present as a concomitant condition in almost 60–95% of patients with asthma. A study of approximately 78,000 asthma patients showed that asthma serves as a risk factor for lung cancer among both males (standardized incidence ratio [SIR]: 1.32) and females (SIR: 1.66).^[8–10]^ Another recent study also reported an elevated risk for squamous cell LC and small cell lung cancer, 1.69, and 1.71 times, respectively, among bronchial asthma patients.^[11]^

As AR depicts an elevated risk for asthma, and asthma represents an elevated risk of lung cancer, in this systematic review and meta-analysis study, we observed whether there was any association between lung cancer and AR. AR is the most common form of allergic condition, and lung cancer is also the most common cancer with the highest mortality rate; therefore, assessing the correlation between these conditions is important.

## 2. Methods

### 2.1. Search strategy

The Preferred Reporting Items for Systematic Review and Meta-Analysis Guidelines were followed to design the methodology for this systematic review.^[12]^ Three databases were searched to identify relevant studies: PubMed, Google Scholar, and ScienceDirect. An advanced and Expert search strategy was used with the terms “Allergic Rhinitis” OR “Hay Fever” OR “Pollinosis” AND “Lung Cancer” followed by “Title and abstract” for PubMed and “Title, abstract and keywords” for ScienceDirect. The “allintitle” option was used on Google Scholar. The last date for search was September 2023. The whole search was done without any time restriction. Boolean operators were used for different database searches. The search strategy is presented in Supplementary Table 1, http://links.lww.com/MD/M510.

### 2.2. Eligibility criteria

Rigorous and specific searches were performed for articles on AR and lung cancer, using specific keywords and search strategies. However, because of the large number of articles, we excluded systematic or comprehensive reviews, correspondence, letters, and other articles that did not align with our interests. Duplicate articles from different databases were excluded. Only articles written in English were included in this study. After excluding the articles, case-control studies that reported the number of AR cases between lung cancer and non-lung cancer groups (as controls) were considered for this systematic review and meta-analysis. Studies were included irrespective of age, sex, race, or country. Studies were excluded if no conclusive association was reported between AR and lung cancer.

### 2.3. Data extraction

Based on the eligibility criteria, data extraction was done carefully from the included studies by the authors independently. The following information from each eligible study was noted in the Microsoft Office Excel Spreadsheet: First author, year, country, number of cases and controls, age of cases and controls, confirmatory method of both AR and lung cancer, and patient recruitment period.

### 2.4. Quality assessment

Quality assessment of the eligible studies was performed based on the Joanna Briggs Institute critical appraisal tool for case-control studies. Any discrepancies were resolved through discussions among the authors. If <50% of queries were satisfied from the checklist, those studies were considered as low-quality studies; however, if 50% to 70%, and >70% of queries were satisfied, those studies were regarded as moderate- and high-quality studies, respectively.^[13]^

### 2.5. Data analysis

Odds ratio (OR) was used to evaluate whether AR serves as a risk factor for lung cancer, and a random effects model was used for the analyses. Statistical significance was set at *P* < .05. The heterogeneity of the included studies was determined using *I*^2^ statistics (*I*^2^ > 75% implies substantial heterogeneity). RevMan (version 5.4) software was used for meta-analysis.

## 3. Results

### 3.1. Study selection

**Figure 1.**
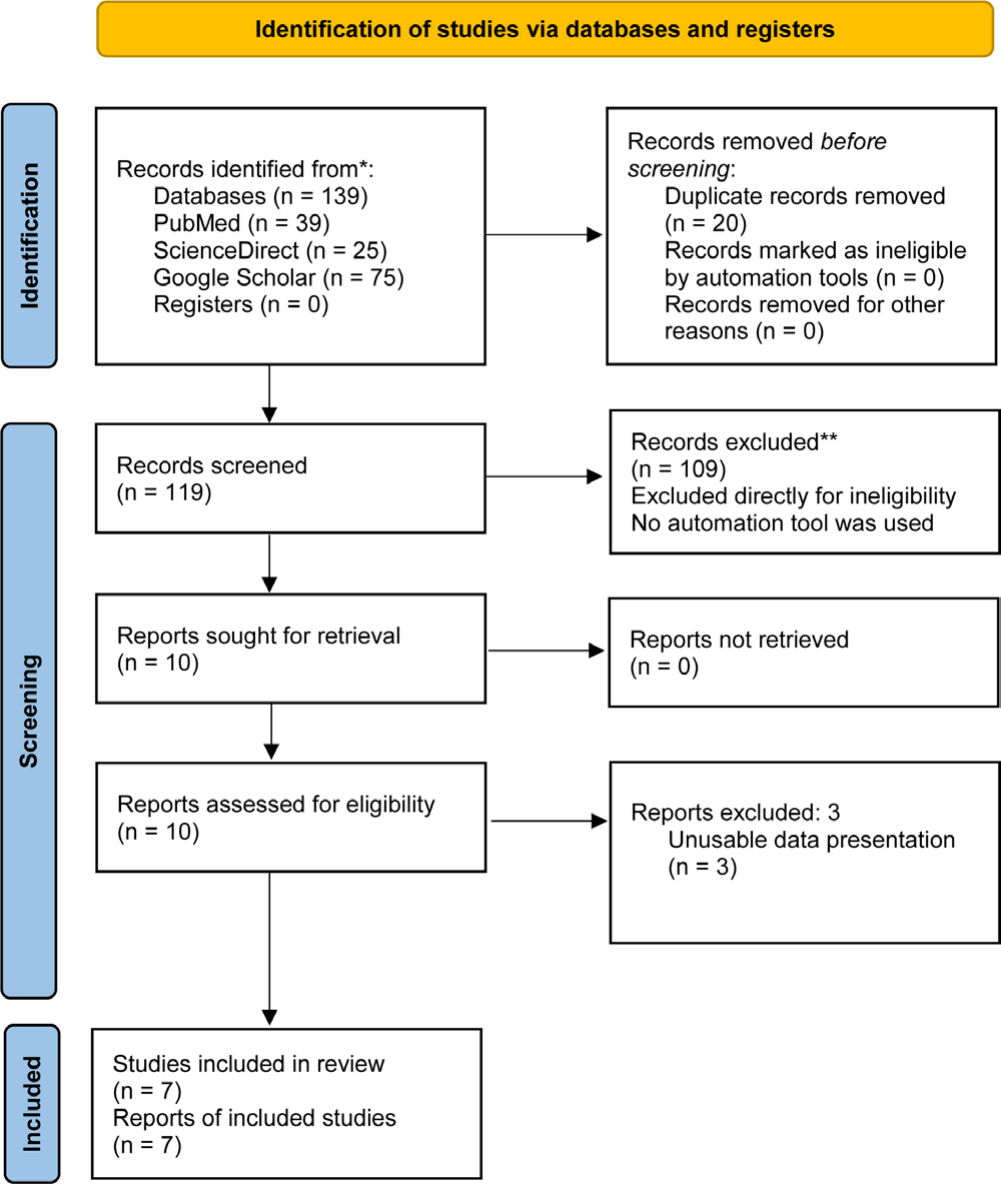
PRISMA flow diagram. PRISMA = Preferred Reporting Items for Systematic Review and Meta-Analysis.

A total of 139 articles from 3 scientific databases: PubMed (39), ScienceDirect (25), and Google Scholar (75), were identified through the search strategy. After removing irrelevant and duplicated studies (129), 10 case-control studies were identified. Those 10 studies were assessed, but 3 had unusable data formats and were subsequently excluded. Therefore, 7 studies were included in the systematic review and meta-analysis^[14–21]^ (Fig. 1).

### 3.2. Quality assessment

The quality assessment of the studies determined that all included studies were of moderate or high quality. No low-quality studies were found; out of 7 eligible studies, 6 were of high quality, and only one study (Spitz et al 2011) was identified as a moderate-quality study, satisfying 60.0% of positive answers (Supplementary Table 2, http://links.lww.com/MD/M511).

### 3.3. Study characteristics

Of the 7 studies that were considered eligible, 5 were conducted in the United States, whereas the remaining 2 were from Canada and Germany. Six studies comprehensively demonstrated the characteristics of both cases and controls, and all reported a mean age of above 60 years. The inclusion period for the study participants ranged from 1990 to 2003. One study was conducted only among the female population,^[16]^ and 2 studies had a higher proportion of females in both cases and controls (Table 1).^[15,18]^ All studies confirmed lung cancer cases histologically; however, AR was confirmed by self-confirmation by interview, except in one study that diagnostically estimated the IgE level for the confirmation of AR.^[19]^ Overall, the study included data from 4724 cases and 9059 controls; however, 47.2% of non-lung cancer subjects were included by Wang et al.

**Table 1:** Characteristics of the included studies.

Study ID	Country	Number of cases (female)	Number of controls (female)	Age mean ± SD (cases) yr	Age mean ± SD (controls)	Lung cancer confirmation	Allergic rhinitis confirmation	Patient recruitment period
Osann et al 2000 ^[16]^	United States	98 (98)	204 (204)	61.7	62.6	Histologically	Self-report (Interview)	July 1990-June 1993
Schabath et al 2005 ^[17]^	United States	1553 (736)	1375 (675)	61.8 ± 10.7	61.2 ± 9.7	Histologically	Self-report (Interview)	August 1995-June 2003
Gorlova et al 2006 ^[15]^	United States	280 (189)	242 (177)	60.2 ± 12.7	61.9 ± 10.9	Histologically	Self-report (Interview)	September 1995-December 2003
Wang et al 2006^[19]^	Germany	196 (UD)	4271 (UD)	63.8 ± 6.8	61.8 ± 6.6	Histologically	IgE diagnosis	June 2000-December 2002
Wu et al 2007^[20]^	United States	977 (453)	977 (453)	62.0 ± 9.8	61.2 ± 9.6	Histologically	NR	NR
Spitz et al 2011^[18]^	United States	451 (304)	508 (318)	61.6 ± 13.0	56.6 ± 13.1	Histologically	NR	NR
El-Zein et al 2014^[14]^	Canada	1169 (458)	1486 (606)	Men: 64.1 ± 7.9 Women: 61.5 ± 9.3	Men: 65.0 ± 7.6 Women: 61.7 ± 9.3	Histologically	Self-report (Interview)	January 1996-December 1997

NR = not reported, UD = undetectable.ss

### 3.4. Outcome

**Figure 2.**
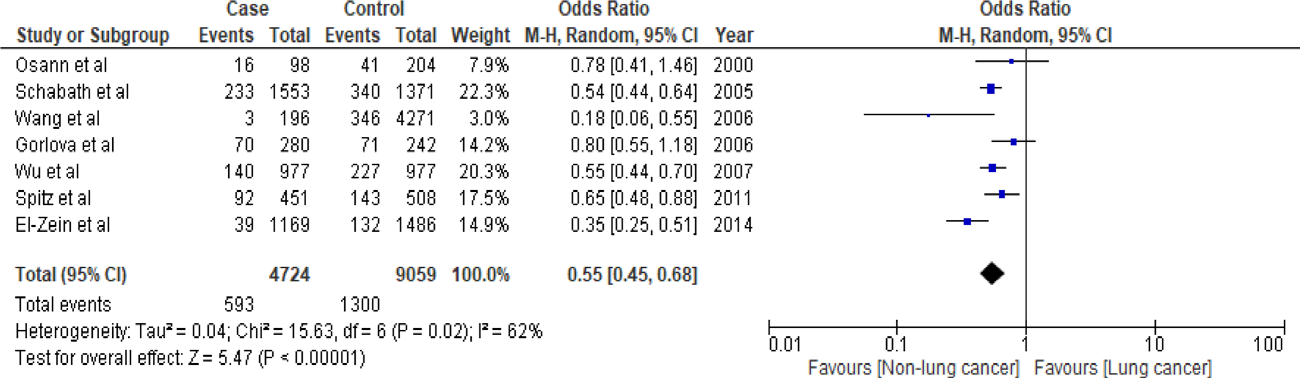
Forest plot with odds ratio (OR) using random effects model.

Pooled analyses were conducted using the random effects model (RE) to determine the OR. The analyses illustrated a protective association between AR and lung cancer (OR: 0.55; 95% CI: 0.45–0.68; *P* value < .00001). The *I*^2^ value was 62%, indicating that the analyses produced a moderate risk of bias. However, the protective association was statistically significant, as shown by the *P* value (*P* < .05) (Fig. 2).

## 4. Discussion

AR causes inflammation of nasal membranes and eyes. Common symptoms experienced by patients include a runny nose, sneezing, nasal itching, congestion, redness, and itching in the eyes. The level of serum IgE is primarily used to diagnose AR.^[21]^ There are similarities between the pathophysiology of AR and asthma, specifically in terms of inflammatory patterns, mediators, and adhesion molecules. Treatment of AR with antihistamines has also led to improved asthma outcomes. Interestingly, up to 94% of patients with allergic asthma have a history of AR according to epidemiological data.^[22]^ Since AR is associated with asthma and asthma is associated with lung cancer, we assumed that AR might have an indirect or direct association with lung cancer. Therefore, in this systematic review and meta-analysis, we tried to reveal this interesting question clinically based on epidemiological data.

Connections between IgE immune responses and defense against cancers were first reported in the 1960s,^[23]^ and numerous studies have pointed to IgE involvement in natural antitumor immunosurveillance.^[24,25]^ Significant epidemiological data suggest that IgE, allergy, and atopy may play protective roles against specific tumor types, with an increased risk associated with IgE immunodeficiency.^[26]^

Experimental studies indicate that IgE antibodies against overexpressed tumor antigens are more efficient in triggering antibody-dependent cellular cytotoxicity/phagocytosis reactions than other immunoglobulin types. IgE can nonspecifically bind to cancer cells, promoting the establishment of tumor-specific immune memory and serving as a potent adjuvant. In addition, immune cells such as eosinophils, macrophages, and mast cells attached to cytosolic IgE could serve as potent antitumor effectors by releasing toxic mediators, proteases, cytokines, and chemokines that cause target cell lysis. These characteristics improve immune cell recruitment, surveillance, and antitumor function.^[27,28]^ A prophylaxis hypothesis suggests that allergic symptoms, such as coughing and sneezing in response to allergen exposure, protect themselves and expel potential carcinogens before they can cause harm.^[26]^

A Canadian study by El-Zein et al found that asthma, eczema, and hay fever are less likely to be associated with lung cancer. AR had the strongest inverse correlation (OR: 0.37), followed by eczema (OR: 0.73), and asthma had a nearly neutral correlation (OR: 0.90); however, it did not favor lung cancer.^[14]^ A study of 138,723 diagnostically confirmed AR patients also showed a SIR of 0.78 in lung cancer, exhibiting a protective association in lung cancer.^[29]^ Our pooled OR was only 0.55; therefore, according to population-based studies, AR patients have a lower risk of lung cancer.^[29]^

Two large-scale studies, each conducted among more than 100,000 patients, reported a higher SIR of kidney, brain, and nasal cavity cancer among AR patients; the available pieces of evidence also did not report any positive association between AR and lung cancer.^[29,30]^ Another study by Kantor et al demonstrated a negative association between allergies (including hay fever, skin allergy, and food allergy) and lung cancer in approximately 50,000 people, but it did not specify risk among hay fever patients separately.^[31]^

All our analyses demonstrated that AR has a protective association with lung cancer, based on available population-based studies. Mechanistically, some pathological insights have revealed why IgE elevation is helpful for tumor suppression. However, studies focusing only on AR have not yet been conducted.

Our study has several strengths. To the best of our knowledge, this is the first meta-analysis to assess the epidemiological association between AR and lung cancer through meta-analysis. The analyses generated low to moderate heterogeneity; therefore, the risk of publication bias was low.

Our study had some limitations. This study included a population from only 3 countries, and the most recent patient recruitment year was 2003. A small number of articles were available for the meta-analysis. The analysis included only case-control studies; cohort studies were not included, as the studies we found in our literature review did not fulfill the inclusion criteria for systematic review. Only one study reported the diagnosis of AR based on IgE levels, while 2 studies did not report any diagnostic method, and in 4 studies, the AR was self-reported. Missing standard diagnostic criteria for AR is another limitation.

### 4.1. Future directions

The strength of the association between AR and lung cancer may differ depending on study location. Additional epidemiological studies are needed to further investigate the presence of AR in patients with lung cancer. Additionally, it is essential to gain pathophysiological insights into whether AR plays a role in the prevention of lung cancer. If more case-control studies are conducted, an updated systematic review and meta-analysis is encouraged to be conducted to incorporate new findings.

## 5. Conclusion

AR is characterized by higher IgE levels, and our study showed a strong protective association between AR and lung cancer. There is experimental evidence in the literature that a higher IgE level has an inverse relationship with lung cancer, and epidemiological studies have also reported an inverse association between allergies and several types of cancer. As no population-based studies were found after 2003, following a very comprehensive search strategy in our study, recent epidemiological studies across different parts of the world are necessary to validate our findings. Further epidemiological studies are required to assess the exact correlation between AR and lung cancer.

## Acknowledgments

Direct technical help in the form of statistics/data manipulation was provided by Google Scholar, PubMed, and Science Direct: Furthermore, indirect assistance was provided by SciFinder® and Sci-hub. Indirect assistance was provided by Google Scholar, PubMed, and Science Direct SciFinder® and Sci-hub in the form of literature.

## Author contributions

**Conceptualization:** Qudsia Umaira Khan, Sohail Khan Raja.

**Data curation:** Qudsia Umaira Khan, Muneeb Ur Rehman, Sabahat Tasnem.

**Formal analysis:** Amna Akbar, Sabahat Tasnem.

**Methodology:** Mohammad Ali Arshad Abbasi, Rubina Rafique Shiekh.

**Resources:** Munazza Nazir, Sarosh Khan Jadoon.

**Software:** Sabahat Tasnem, Sarosh Alvi.

**Supervision:** Sohail Khan Raja.

**Writing – original draft:** Mohammad Ali Arshad Abbasi, Amna Akbar.

**Writing – review & editing:** Qudsia Umaira Khan, Munazza Nazir, Sarosh Khan Jadoon.

## Supplementary Material
