# Supplementary material for: Correlation between allergic rhinitis or hay fever and lung cancer: A systematic review and meta-analysis
Source: Medicine (Baltimore). 2024 May 17;103(20):e38197. doi: 10.1097/MD.0000000000038197 (PMC11098191; doi:10.1097/MD.0000000000038197)
Supplement: Supplementary file 1 [file medi-103-e38197-s001.docx]

**Supplementary Table 1: Search strategy**

| Databases | Search Strategies |
| --- | --- |
| PubMed | (((allergic rhinitis[Title/Abstract]) OR (hay fever[Title/Abstract])) OR (pollinosis[Title/Abstract])) AND (lung cancer[Title/Abstract]))) |
| Google Scholar | allintitle: allergic rhinitis AND lung cancer |
|  | allintitle: hay fever AND lung cancer |
|  | allintitle: pollinosis AND lung cancer |
| ScienceDirect | Title, abstract, keywords: allergic rhinitis, lung cancer |
|  | Title, abstract, keywords: hay fever, lung cancer |
|  | Title, abstract, keywords: pollinosis, lung cancer |
