# Supplementary material for: Correlation between allergic rhinitis or hay fever and lung cancer: A systematic review and meta-analysis
Source: Medicine (Baltimore). 2024 May 17;103(20):e38197. doi: 10.1097/MD.0000000000038197 (PMC11098191; doi:10.1097/MD.0000000000038197)
Supplement: Supplementary file 2 [file medi-103-e38197-s002.docx]

| **Table S1: Quality Assessment of the Eligible Studies** | | | | | | | | | | | |
| --- | --- | --- | --- | --- | --- | --- | --- | --- | --- | --- | --- |
| **Study ID** | **1** | **2** | **3** | **4** | **5** | **6** | **7** | **8** | **9** | **10** | **Total Score %** |
| Osann *et al.*  2000 | Y | Y | Y | Y | Y | Y | Y | U | Y | Y | 90.0 |
| Schabath *et al.*  2005 | Y | Y | Y | Y | Y | Y | Y | U | Y | Y | 90.0 |
| Gorlova *et al.*  2006 | Y | Y | Y | Y | Y | Y | Y | U | Y | Y | 90.0 |
| Wang *et al.*  2006 | Y | Y | Y | Y | Y | Y | Y | Y | Y | Y | 100.0 |
| Wu *et al.*  2007 | Y | Y | Y | Y | Y | Y | Y | U | U | Y | 80.0 |
| Spitz *et al.*  2011 | Y | Y | Y | Y | Y | N | N | U | U | Y | 60.0 |
| El-Zein *et al.* 2014 | Y | Y | Y | Y | Y | Y | Y | U | Y | Y | 90.0 |
| Questions:   1. Were the groups comparable other than presence of disease in cases or absence of disease in controls? 2. Were cases and controls matched appropriately? 3. Were the same criteria used for identification of cases and controls? 4. Was exposure measured in a standard, valid and reliable way? 5. Was exposure measured in the same way for cases and controls? 6. Were confounding factors identified? 7. Were strategies to deal with confounding factors stated? 8. Were outcomes assessed in a standard, valid and reliable way for cases and controls? 9. Was the exposure period of interest long enough to be meaningful? 10. Was appropriate statistical analysis used? | | | | | | | | | | | |
| Y = Yes = 1, N = No = 0, U = Unclear = 0 | | | | | | | | | | | |
